# Systematic analysis of the RGS2 degron reveals characteristics of substrate recognition by the F-box protein FBXO44

**DOI:** 10.1016/j.jbc.2025.110757

**Published:** 2025-09-22

**Authors:** Harrison J. McNabb, Eugene Cho, Mary Pitman, Phillip S. Rushton, David Mobley, Benita Sjögren

**Affiliations:** 1 Department of Medicinal Chemistry and Molecular Pharmacology, Purdue University, West Lafayette, Indiana, USA; 2 Department of Pharmaceutical Sciences, University of California, Irvine, Irvine, California, USA; 3 Robert A. Mah Molecular Innovation Center, University of California, Irvine, Irvine, California, USA; 4 Department of Biological Chemistry, University of California, Irvine, Irvine, California, USA

**Keywords:** regulator of G protein signaling, protein degradation, E3 ubiquitin ligase, peptide array, molecular modeling

## Abstract

Regulator of G protein signaling 2 (RGS2) negatively modulates signaling downstream of G protein–coupled receptors by accelerating GTP hydrolysis at Gα subunits of heterotrimeric G proteins. Decreased RGS2 levels are implicated in numerous diseases, including cardiovascular disease and asthma. Thus, identifying selective means of enhancing RGS2 protein levels would be a viable therapeutic strategy. RGS2 is rapidly degraded through the ubiquitin–proteasomal pathway, and we previously identified F-box only protein 44 (FBXO44) as the substrate recognition component of the E3 ligase responsible for facilitating RGS2 degradation. As such, the RGS2–FBXO44 interaction is a potential target for pharmacological intervention. Detailed information on the FBXO44 recognition site (degron) in RGS2 will aid in structure-based small-molecule inhibitor design, as well as in identifying additional FBXO44 targets, which would help predict possible side effects of targeting this interaction. Thus, the goal of this study was to dissect the molecular properties for FBXO44 binding of the RGS2 degron. We used a peptide array utilizing systematic residue substitution, combined with AlphaFold modeling and molecular dynamics simulations, to identify several amino acid changes that altered binding both positively and negatively. Finally, we experimentally confirmed our results in cells through coimmunoprecipitation and proteasomal inhibition, using full-length RGS2. Altogether, these results provide structural insights into RGS2–FBXO44 binding, which will aid in structure-guided drug discovery efforts. It also provides a framework for building a consensus recognition motif for FBXO44, which could aid in identifying more substrates for this understudied F-box protein.

Since their discovery in the mid-1990s, regulators of G protein signaling (RGS) proteins have emerged as key negative modulators of signaling through G protein–coupled receptors that show promising potential as novel drug targets (1, 2, 3). Through their canonical GTPase accelerating protein activity, as well as other functions, often mediated by the presence of additional domains, RGS proteins fine-tune both G protein–dependent and –independent signaling pathways. Altered RGS protein function is associated with numerous pathologies, including cardiovascular and neurological conditions (4, 5, 6, 7, 8). In many cases, decreased RGS action is associated with disease progression, and strategies that promote RGS protein activity would be needed to provide a beneficial clinical outcome. One such example is represented by RGS2. Low RGS2 protein levels are associated with a number of pathologies, including hypertension, heart failure, and asthma (6, 7). RGS2 is uniquely selective for Gα_q_, partly explaining its effects on blood pressure regulation. The hypertensive phenotype displayed that RGS2^−/−^ mice is linked to augmented responses to Gq-linked vasoconstrictors, such as angiotensin II (9). Impact on Gq signaling can also partly explain effects of RGS2 in the bronchi; however, noncanonical regulation of Gs-mediated signaling can also explain the effects of RGS2 on airway constriction (10). We previously showed that pharmacological RGS2 protein stabilization is protective in a model of cardiac injury *in vivo* (11). Hence, targeting the mechanism(s) regulating RGS2 protein levels represents a novel strategy in diseases associated with low RGS2 protein levels.

RGS2 is rapidly and constitutively degraded by the ubiquitin–proteasomal system (12, 13). Dysfunction of this crucial system can lead to accumulation of misfolded or aged proteins, cell cycle arrest, and uncontrolled cell proliferation (14, 15, 16, 17), driving various pathologies, including multiple cancers and cardiovascular disease (18, 19). Proteasome inhibitors, such as bortezomib (Velcade) and carfilzomib, are used in the treatment of certain blood cancers (20, 21). However, the severe side effects associated with these drugs, including neuropathies, fatigue, and anemia, limit their use. Members of the large family (>700) of E3 ligases confer specificity within the ubiquitin–proteasomal system (14, 15), and targeting individual E3 ligase–substrate pairs shows promise as a more selective strategy to reduce the side effects (22, 23). We previously identified F-box only protein 44 (FBXO44) as a component of a Cullin-RING E3 ligase (CRL) that recognizes and targets RGS2 for degradation (24). F-box proteins show promise as drug targets, not only for several types of cancer but also for neurological and cardiovascular diseases. Of the 69 F-box proteins known, FBXO44 is one of the least characterized. Only two other substrates have been identified (25, 26), and the mechanism of substrate recognition is unknown. While FBXO44 is closely homologous to a family of glycan-binding F-box proteins, it does not bind the same substrates (27).

We previously identified the region in RGS2, or degron, which gets recognized by and binds FBXO44 (28). This region is located close to the RGS2 N terminus (Met^5^–Met^16^), and we demonstrated that a peptide consisting of these residues could block RGS2–FBXO44 coimmunoprecipitation (co-IP) *in vitro* (28). These studies identify the RGS2–FBXO44 interaction as a potential point of intervention for small-molecule RGS2 stabilizers, and we recently identified several such compounds in a high-throughput screen (29). However, several questions remain. First, direct interaction between RGS2 and FBXO44 has not yet been established. Second, the sequence of the RGS2 degron is not conserved in the other two known substrates, BRCA1 and the pregnane X receptor (25, 26). The degron in BRCA1 is only narrowed down to the first 167 N-terminal residues (25), and the exact sequence of the degron we identified in RGS2 is absent within this region. However, identifying what chemical properties are important for FBXO44-mediated degradation of RGS2 may provide insight into predicting the degron of potential FBXO44 substrates identified in the future. Understanding the biochemical mechanism by which FBXO44 recognizes RGS2 for proteasomal degradation will also enable rational drug design of protein–protein interaction (PPI) inhibitors. Thus, in the current study, we used both *in vitro* and *in silico* methods to identify the important residues for FBXO44 binding of the RGS2 degron and examined how mutation of these residues affects RGS2 degradation in cells.

## Results

### An RGS2 degron peptide binds FBXO44^FBA^*in vitro*

**Figure 1:**
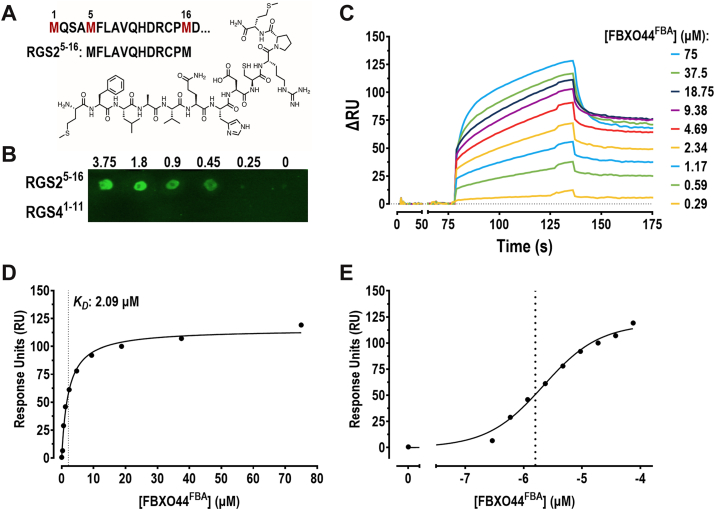
**A peptide representing the degron in RGS2 binds FBXO44^FBA^*in vitro*.***A,* sequence and schematic of the RGS2^5–16^ peptide. *B,* a representative dot blot showing binding of FBXO44^FBA^ to immobilized RGS2^5–16^ peptide. Peptide was spotted onto PVDF membrane at the indicated concentrations and then incubated with 2 μg/ml purified FLAG-FBXO44^FBA^, probed with anti-FLAG primary, and imaged. A peptide corresponding to the first 11 residues in RGS4 (RGS4^1–11^) was used as a negative control. *C,* SPR sensorgram of specific FBXO44^FBA^ binding to immobilized RGS2^5–16^ peptide. Fc1 was immobilized with a blank buffer, and Fc2 was immobilized with RGS2^5–16^ peptide. FBXO44^FBA^ was flowed over the chip starting at the lowest concentration of 0.29 μM and repeated through to the highest concentration at 75 μM. The RU at equilibrium was subtracted from the nonspecific binding detected in Fc1 and measured for each concentration. Total and nonspecific binding sensorgrams are displayed in Figure S1. *D* and *E,* nonlinear fit of FBXO44^FBA^ saturation binding, *K*_*D*_ is displayed and represented as a *vertical dotted line*. FBXO44, F-box only protein 44; PVDF, polyvinylidene difluoride; RGS2, regulator of G protein signaling 2; RU, response unit; SPR, surface plasmon resonance.

We previously demonstrated that RGS2 associates with FBXO44 through a stretch of residues in its N terminus and that this binding promotes RGS2 ubiquitination and subsequent proteasomal degradation (28). However, evidence of a direct interaction between RGS2 and FBXO44 has, until now, not yet been established. Thus, we first aimed to verify that purified FBXO44 would bind the degron sequence *in vitro*. As we previously showed that the F-box associated (FBA) domain is sufficient to bind RGS2 in cell lysate (24), we expressed and purified only this domain for all *in vitro* studies. This also circumvented the issue that full-length FBXO44 (as well as all other F-box proteins) is very difficult to express in the absence of an adaptor protein, such as Skp1 (30). To test the ability of the FBA domain to bind the RGS2 degron specifically, a peptide corresponding to residues Met^5^–Met^16^ (MFLAVQHDRCPM; herein referred to as RGS2^5–16^; Fig. 1*A*) was dotted onto a polyvinylidene difluoride (PVDF) membrane at increasing amounts (Fig. 1*B*). As a negative control, we used a peptide from the closely related RGS4 (RGS4^1–11^; MCKGLAALPAT), which, while also subject to proteasomal degradation, utilizes a different pathway and does not coimmunoprecipitate with FBXO44 (24). The membrane was then incubated with FLAG-tagged FBXO44 FBA domain (FBXO44^FBA^) and subjected to immunoblotting using a FLAG antibody. We observed a dose-dependent increase in FLAG immunoreactivity using the RGS2^5–16^ peptide, with no signal detected using the RGS4^1–11^ peptide (Fig. 1*B*). This indicates that FBXO44^FBA^ can bind the RGS2 degron *in vitro* and that the interaction is selective for RGS2 over the RGS4 N terminus. To get an estimate of the binding affinity between FBXO44^FBA^, we next performed surface plasmon resonance (SPR) with the RGS2^5–16^ peptide anchored on the chip and FBXO44^FBA^ as the analyte. Using increasing concentrations of FBXO44^FBA^, we determined that the RGS2 degron binds the FBA domain with a *K*_*D*_ value of ∼2.1 μM, providing further evidence that the FBXO44^FBA^ is capable of binding the RGS2^5–16^ peptide *in vitro* with reasonable affinity (Fig. 1, *C*–*E*).

### A peptide array reveals key residues important for the RGS2–FBXO44 interaction

**Figure 2:**
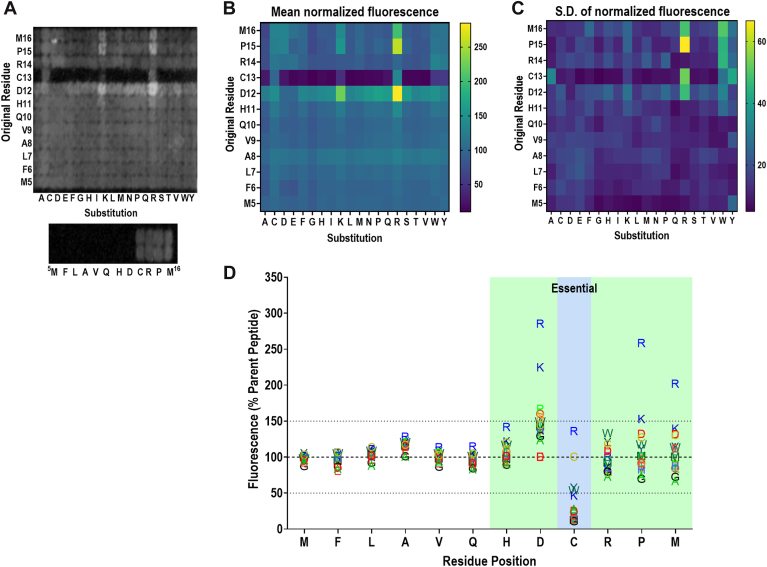
**Systematic mutation of the RGS2 degron alters FBXO44^FBA^ binding.** A custom microarray (PEPperPRINT) was designed where each position of the RGS2^5–16^ peptide was substituted for each natural amino acid. We also included truncated peptides on the array. This was incubated with purified FBXO44^FBA^ and probed with α-FLAG. *A,* a representative scan of a peptide microarray (*top*) and a truncated peptide (*bottom*). *B,* heatmap of mean fluorescence of quantified microarray scans. The scale represents the mean fluorescence in percentage points across all replicates, normalized to the signal obtained with the original residue at each position. *C,* heatmap of standard deviation of quantified microarray scans. The scale represents the standard deviation in percentage points across all replicates. *D,* the mean of quantified results displayed as the one-letter abbreviation of the amino acid substitution. Residues are color-coded based on properties: *blue*, positive charge; *red*, negative charge; *orange*, uncharged polar; *green*, hydrophobic; *yellow*, Cys (C); *bright green*, Pro (P); and *black*, Gly (G). *Green background shading* indicates residues with statistical significance, and *blue background shading* indicates that the residue is essential for FBXO44^FBA^ binding. Results of three independent experiments with three technical replicates each. For a statistical significance summary, see Table S1. FBXO44, F-box only protein 44; RGS2, regulator of G protein signaling 2.

**Figure 3:**
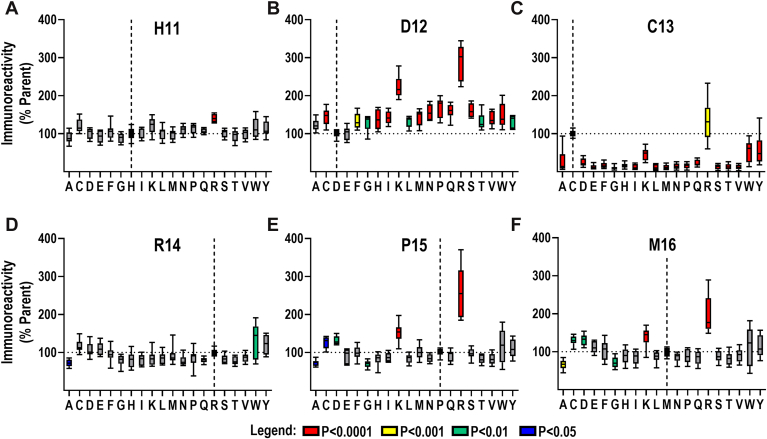
**Effects of RGS2^5–16^ peptide residue changes on FBXO44^FBA^ binding.** Data are presented as % FLAG immunoreactivity, as compared with the parent peptide residue. Positions in *A*–*F* represent residues where at least one substitution resulted in a significant change in FBXO44^FBA^ binding (highlighted in *blue* and *green* in Fig. 2*C*). The original residue is marked in each graph with a *vertical dotted line*. *Boxes* extend from the 25th to 75th percentile with a *line* marking the median. *Whiskers* extend to the minimum and maximum. Results of three independent experiments with three technical replicates each. Data were analyzed by two-way ANOVA, with Tukey's *post hoc* test for pairwise comparison (simple effect within columns). Statistical significance is summarized in Table S1 and color-coded according to the legend. FBXO44, F-box only protein 44; RGS2, regulator of G protein signaling 2.

Having demonstrated that the FBXO44^FBA^ binds the RGS2^5–16^ peptide *in vitro*, we sought to probe the sequence to identify important residues and properties for binding. To do so, we utilized a custom peptide microarray (PEPperPRINT GmbH). The design of this array was twofold: (1) substitution at each position with every other natural eukaryotic amino acid; (2) truncation of the peptide from the C terminus. We assessed the binding of FLAG-FBXO44^FBA^ to the array utilizing fluorescence immunoreactivity corresponding to the amount of FLAG-FBXO44^FBA^ bound. In agreement with previous studies by us and others (28, 31), changes within the first, predominantly hydrophobic region of the peptide, corresponding to residues 5 to 10 in RGS2, had no significant effect on FBXO44^FBA^ binding, indicating that these residues are not participating in the RGS2–FBXO44 interaction (Fig. 2). However, changes within residues His^11^–Met^16^ resulted in both statistically significant increases and decreases in fluorescence signals (Figs. 2 and 3). Substitutions at His^11^ and Arg^14^ had modest effects, with only one or two substitutions for each reaching significance (H11R, R14A, and R14W; Fig. 3, *A* and *D*). Substituting Pro^15^ with either Ala or Gly slightly, but significantly, impaired FBXO44^FBA^ binding, possibly because of increased flexibility in the amino backbone (Fig. 3*E*). The effects at Cys^13^ were the most pronounced, and changing Cys^13^ to any other amino acid (except for Arg) significantly reduced FBXO44^FBA^ binding (Fig. 2, 3*C*), indicating that Cys^13^ is essential for the RGS2–FBXO44 interaction. Substitutions at Asp^12^ were not only tolerated but also seemed to improve binding in every case (Fig. 3*B*). This, combined with the fact that substituting any residue in His^11^–Met^16^ with Arg, and in most cases with Lys, improved FBXO44^FBA^ binding, indicating the presence of negative charges at the binding interface. A summary of statistically significant changes is presented in Table S1.

### AlphaFold modeling of the RGS2–FBXO44 interaction supports cysteine-dependent binding

**Figure 4:**
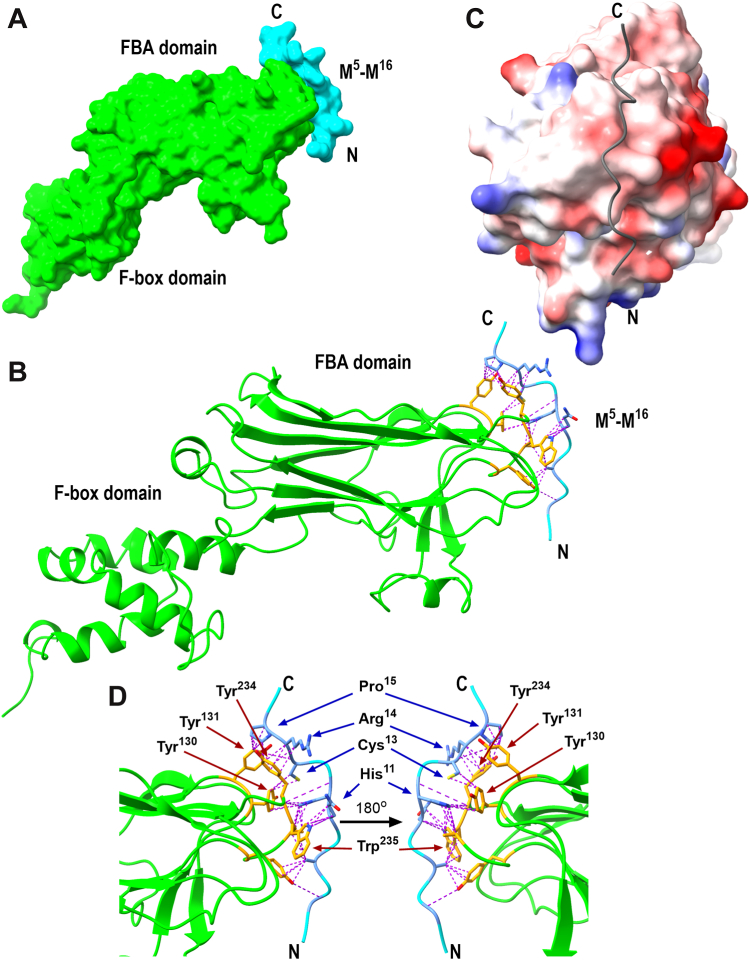
**AlphaFold-predicted structure of the RGS2^5–16^–FBXO44 interaction.***A,* overview of the AlphaFold best model for the sequences for FBXO44 (UniProt: Q9H4M3-1) and RGS2^5–16^. FBXO44 displayed in *green*, RGS2^5–16^ in *cyan* with N and C termini labeled. RGS2^5–16^ is predicted to bind the FBA domain in a region homologous to the substrate-binding loops of other FBX proteins (35). *B,* Side view of the predicted ribbon structure with contact residues predicted by ChimeraX displayed as *sticks* with *purple* pseudobonds. FBXO44 contact residues are colored *orange* and RGS2 ones are a *muted blue*. *C,* electrostatic map of the RGS2–FBXO44 interaction interface. FBXO44 is displayed as a surface map colored according to charge (*red* being more negatively charged, *blue* more positively charged) and RGS2^5–16^ is depicted as a *gray loop* with termini labeled. The predicted interaction surface on FBXO44 is predominantly slightly negative. *D,* close-up of the RGS2^5–16^–FBXO44 interaction interface. RGS2 residues of interest are labeled with *blue arrows*, whereas predicted contact residues of FBXO44 are labeled with *red arrows*. FBXO44, F-box only protein 44; RGS2, regulator of G protein signaling 2.

To complement our *in vitro* studies, we next used *in silico* methods to further analyze the RGS2–FBXO44 interaction. As there is no published structure of RGS2 that includes the N terminus, we decided to use predictive computational tools to construct a potential model of the interaction site. We utilized AlphaFold 3 and UCFS ChimeraX molecular visualization and analysis software (32, 33, 34), inputting the protein sequences for FBXO44 (UniProt: Q9H4M3-1) and the RGS2^5–16^ peptide (Fig. 4). No additional guidance was introduced to the model other than the raw sequences. The resulting best model successfully folded FBXO44 into the correct structure, as compared with the published crystal structure of FBXO44 in complex with Skp1 (3WSO; (35)). The model docked the RGS2^5–16^ peptide onto the β–β loops of the FBA domain. This mode of interaction is homologous with the mode of substrate binding of other FBA-containing F-box proteins (35) (Fig. 4, *A* and *B*). The predicted RGS2^5–16^—FBXO44 complex also reveals a slightly negative surface on FBXO44 at and near the binding site, which would explain why substitutions with Arg (and Lys) enhanced binding in the peptide microarray (Fig. 4*C*).

The AlphaFold Multimer model predicted His^11^, Cys^13^, Arg^14^, Pro^15^, and Met^16^ of RGS2^5–16^ to be in contact with FBXO44. Substitutions at these residues all displayed some change in FBXO44^FBA^ binding in the peptide microarray (Figure 2, Figure 3), validating that the AlphaFold Multimer model is in line with our experimental results. Cys^13^ is the only residue of RGS2^5–16^ predicted to be in a “pocket” of the FBXO44 surface (Fig. 4*D*), which may explain why Cys^13^ is essential for binding.

ChimeraX also predicted residues in the AlphaFold 3 model on FBXO44 important for the interaction with RGS2^5–16^. Tyr^130^, Tyr^131^, Tyr^234^, and Trp^235^ are all being predicted to be in contact with RGS2. Many of these residues are in close proximity to the residues that form unique intramolecular hydrogen bonds in the Skp1-FBXO44 crystal structure (3WSO). The hydrogen bond network within the β–β loops of the FBA domain is important for substrate binding in the closely related FBXO2 and most likely participates in substrate binding for FBXO44 as well (35), though the substrate binding site in FBXO44 has not been established. Of the residues predicted to form contacts with RGS2^5–16^, Tyr^234^ stood out to be of particular interest. This residue is conserved between FBXO44 and FBXO2 and is one of the residues responsible for substrate binding in FBXO2 (35). In FBXO44, Tyr^234^ participates in hydrogen bonding with Asp^169^ and Cys^170^, stabilizing a unique conformation that does not occur in FBXO2 (35).

### Molecular dynamics simulations reveal key residues of the RGS2–FBXO44 interaction

**Figure 5:**
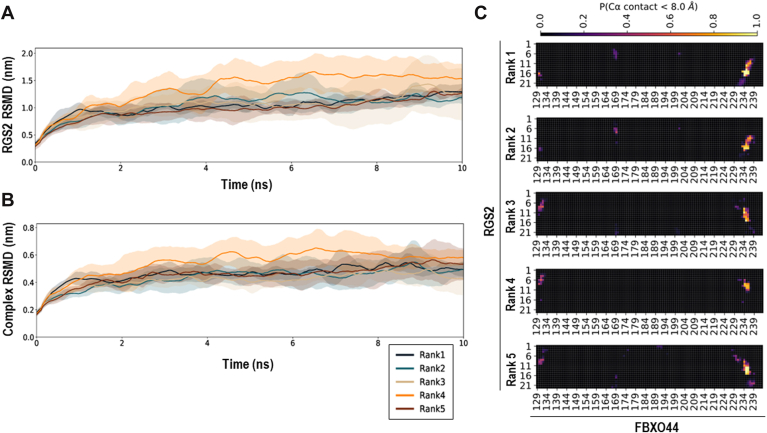
**Molecular dynamics (MD) simulation of the FBXO44^FBA^–RGS2^1–22^ interaction.***A,* average RMSD of the RGS2^1–22^ domain compared with the conformation at the start of MD production runs over four simulation trials. Each color corresponds to a binding pose rank (1–5) as scored by AlphaFold Multimer. The variance in the RMSD is shown by the *shaded regions*, and the average RMSD values are shown by *lines*. *B,* the average RMSD plots of the whole complex formed by FBXO44 and RGS2^1–22^ as plotted with identical methods as in *A*. *C,* contact analysis plots of FBXO44 (F-box only protein 44) shown on the *x*-axes and RGS2^1–22^ shown on the *y*-axes for each of the five ranked poses generated by AlphaFold Multimer. Colors show the probability of a contact being observed in a simulation averaged over four simulation trials. A color of *light yellow* and a score of 1 correspond to a contact being observed between residues in all simulation frames. A color of *black* and a score of 0 correspond to a contact between residues never being observed. FBXO44, F-box only protein 44; RGS2, regulator of G protein signaling 2.

Molecular dynamics (MD) simulations were performed for the top five predicted protein-to-protein conformations generated by AlphaFold Multimer. Each ranked conformation of the binding interaction of FBXO44 to RGS2^1–22^ was simulated for four independent trials using all-atom MD. First, we investigated which binding modes of the PPIs showed the highest stability over all simulation trials. We found that the AlphaFold Multimer–generated poses ranked 1 and 5 showed the highest stability of RGS2^1–22^ binding as demonstrated by both the smallest drift compared with the predicted structure and lower RMSD variances across simulation trials compared with other conformations (Fig. 5*A*). Ranked poses 1, 2, 3, and 5 showed relative RMSD stability for the entire FBXO44–RGS2^1–22^ complex (Fig. 5*B*). From this analysis, we determined that AlphaFold Multimer–generated poses showed sufficient stability in MD when energy was minimized. In addition, the first and fifth ranked poses showed the highest binding stability of RGS2^1–22^ binding to FBXO44.

We next inspected which residues formed stable interactions between FBXO44 and RGS2^1–22^ by performing contact analysis of simulations from each starting pose. Here, we calculated the average contact frequency between interface residues over simulation trials. Consistent with our RMSD stability findings, ranked poses 1 and 5 showed the highest frequency binding of RGS2^1–22^ to FBXO44, with residues forming continuous contacts across all trials (Fig. 5*C*). For the rank 1 pose, FBXO44 Tyr^234^ showed high-frequency interactions with RGS2 residues 15 to 17. In the rank 5 pose, Trp^235^ and Ala^236^ showed stable interactions with RGS2 residue 12 to 14. In all ranked poses, Tyr^234^ formed interactions with RGS2^1–22^. We also previously found that residues FBXO44 Arg^167^ and Asp^169^ were predicted to be contact residues outside MD simulations. Simulations did not show contacts with Arg^167^; however, we saw frequent contacts formed between FBXO44 Pro^168^ and Asp^169^ and RGS2^1–22^.

We next analyzed how RGS2 Cys^13^ interacts with FBXO44. In the first-ranked pose, we found that RGS2 Cys^13^ forms reduced interactions compared with neighboring residues; however, Cys^13^ is centered in a highly stable binding pocket spanning from RGS2 residues 12 to 17. In comparison, in the other most stable conformation, rank 5, we find highly frequent interactions between RGS2 Cys^13^ and FBXO44 residues 235 and 236 (Fig. 5*C*). Taken together, these results support the hypothesis that there may be multiple metastable states for the binding interaction of RGS2 with FBXO44. In addition, RGS2 Cys^13^ mediates one of the most stable binding modes as scored by MD.

### Mutations of key interacting residues impair the RGS2–FBXO44 association in cells

**Figure 6:**
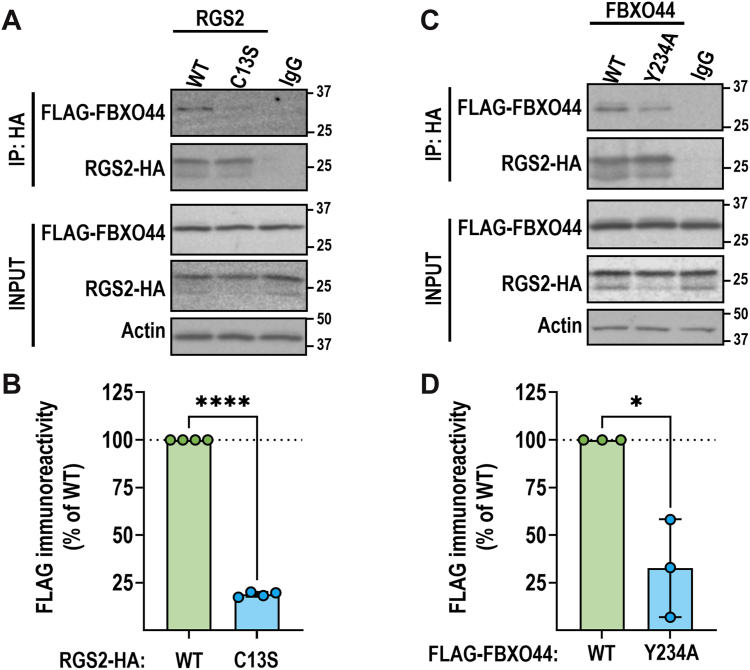
**Point mutations disrupt the RGS2–FBXO44 association.** Co-IP was performed in HEK-293T cells transiently transfected with RGS2-HA and FLAG-FBXO44 (WT and mutants as indicated). *A* and *B,* RGS2^C13S^-HA associates significantly less than RGS2^WT^-HA with FLAG-FBXO44. Representative blot and quantification of four independent experiments. *C* and *D,* FLAG-FBXO44^Y234A^ associates significantly less than FLAG-FBXO44^WT^ with RGS2-HA. Levels of each protein were normalized to input and expressed as % of WT. Representative blot and quantification of three independent experiments. ∗*p* < 0.05; ∗∗∗∗*p* < 0.0001 using Student's unpaired *t* test. Co-IP, coimmunoprecipitation; FBXO44, F-box only protein 44; RGS2, regulator of G protein signaling 2.

Our *in vitro* and *in silico* approaches identified key residues important for the RGS2–FBXO44 interaction. We next sought to confirm that these results would translate to full-length proteins in a cellular context. First, we assessed the effects of two key residues in RGS2 and FBXO44, respectively, on the association between the two proteins. Human embryonic kidney-293T (HEK-293T) cells were transfected with RGS2^C13S^-HA/FLAG-FBXO44^WT^ or RGS2^WT^-HA/FBXO44^Y234A^ and subjected to co-IP utilizing the HA tag on RGS2. Both mutations significantly reduced association between the two proteins (Fig. 6, *A* and *B*), confirming that Cys^13^ in RGS2 and Tyr^234^ in FBXO44 are key contacts for the interaction.

### RGS2^C13S^ displays impaired FBXO44-mediated proteasomal degradation

**Figure 7:**
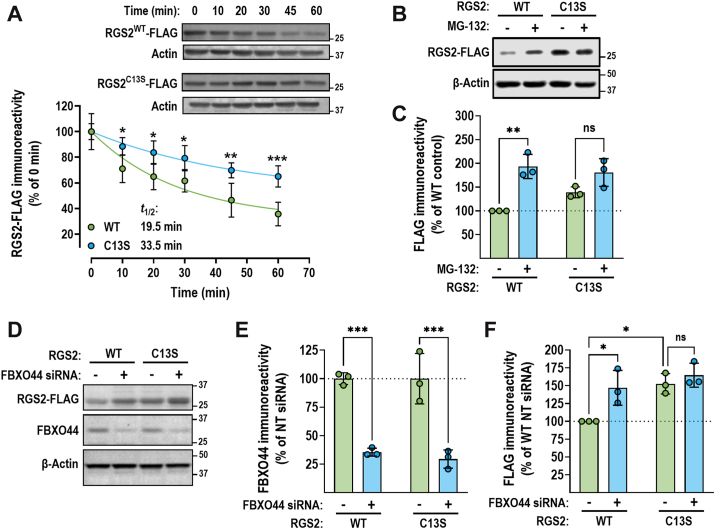
**RGS2^C13S^ is protected from proteasomal degradation.***A,* RGS2^C13S^ is significantly stabilized in a cycloheximide (CHX) chase assay, using transfected HEK-293T cells. Representative Western blot and quantification of three independent experiments. RGS2^WT^ displays a protein half-life (*t*_1/2_) of 19.5 min. In contrast, RGS2^C13S^ displays a protein *t*_1/2_ of 33.5 min, almost double that of RGS2^WT^. ∗*p* < 0.05; ∗∗*p* < 0.01; ∗∗∗*p* < 0.001 using two-way ANOVA (mixed-effect analysis) followed by Bonferroni's *post hoc* test for pairwise comparisons. *B* and *C,* RGS2^WT^ protein levels are significantly enhanced by MG-132 (10 μM, 4 h) in transiently transfected HEK-293T cells. MG-132 treatment does not significantly change RGS2^C13S^ levels. While not statistically significant, there is also an upward trend in basal expression of RGS2^C13S^. Representative Western blot and quantification of three independent experiments. Representative Western blot (*D*), quantification of knockdown efficiency (*E*), and effects on RGS protein levels (*F*) when FBXO44 is knocked down using siRNA. FBXO44 knockdown significantly increases RGS2^WT^ but not RGS2^C13S^ protein levels. Result of three independent experiments. ∗*p* < 0.05; ∗∗*p* < 0.01; ∗∗∗*p* < 0.001 using two-way ANOVA followed by Tukey's *post hoc* test for pairwise comparisons (*C*, *E*, and *F*). FBXO44, F-box only protein 44; HEK-293T, human embryonic kidney-293T cell line; RGS2, regulator of G protein signaling 2.

Inhibiting the RGS2–FBXO44 interaction is predicted to stabilize RGS2 because of reduced proteasomal degradation. Therefore, we assessed the effect of mutating Cys^13^ on RGS2 protein stability. RGS2^C13S^ was significantly stabilized compared with RGS2^WT^, as demonstrated by a cycloheximide (CHX) chase assay (Fig. 7*A*). The RGS2 protein half-life was extended almost twofold (33.5 min [RGS2^C13S^] *versus* 19.5 min [RGS2^WT^]). In our previous studies, RGS2 protein half-life was extended to a similar magnitude when FBXO44 was knocked down using siRNA (24). Next, we assessed the effect of Cys^13^ on proteasomal degradation of RGS2. HEK-293T cells transfected with either RGS2^WT^ or RGS2^C13S^ were treated with the proteasome inhibitor MG-132 (10 μM). Protein levels of RGS2^WT^ were significantly enhanced by MG-132 treatment. In contrast, RGS2^C13S^ levels were not significantly changed, indicating that proteasomal targeting is impaired in this mutant (Fig. 7, *B* and *C*). Furthermore, there was a trend toward RGS2^C13S^, displaying increased protein levels at baseline; however, this increase was not statistically significant. Several other mutations significantly altered RGS2^5–16^–FBXO44^FBA^ binding in the microarray, including D12R, P15R, and P15G. We hypothesized that D12R and P15R will be destabilizing because they both enhanced FBXO44^FBA^ binding, whereas we expected P15G to be stabilizing since it decreased binding. However, none of these mutations resulted in significant changes in basal expression or response to MG-132, as compared with RGS2^WT^ (Fig. S4). Furthermore, while there was a trend toward lower basal expression of RGS2^D12R^, this reduction was not statistically significant.

Finally, to confirm that the altered protein stability displayed by RGS2^C13S^ was dependent on FBXO44, we assessed the effect of siRNA-mediated FBXO44 knockdown on RGS2 protein levels. Using a pool of 4 siRNA oligos (siGENOME SMART Pools; Dharmacon/Revvity), we achieved ∼70% reduction in endogenous FBXO44 protein levels in HEK-293T cells (Fig. 7, *D* and *E*). This resulted in a significant increase in RGS2^WT^ but not RGS2^C13S^ protein levels (Fig. 7, *D* and *F*). In addition, in this series of experiments, RGS2^C13S^ expressed at significantly higher levels than RGS2^WT^ (Fig. 7*F*). Altogether, our results indicate that Cys^13^ is an essential residue for targeting RGS2 for FBXO44-mediated proteasomal degradation.

## Discussion

In this study, we followed up on our previous work to further characterize the RGS2 degron that is recognized by the E3 ligase component FBXO44 (28). While we had previously shown that RGS2 associates with FBXO44 in cells through a stretch of residues near its N terminus, we had not yet demonstrated direct interaction between the two proteins. Here, we provide evidence that the substrate-binding domain of FBXO44, the FBA domain, binds an RGS2^5–16^ peptide both in a dot blot approach and in SPR (Fig. 1). SPR allowed us to calculate a preliminary *K*_*D*_ of 2.1 μM. While this might be considered a moderate affinity interaction, it is within range of what has previously been found for E3 ligase–substrate interactions, which can vary greatly (36). Furthermore, our determined *K*_*D*_ value may not be reflective of the affinity of full-length RGS2 in a cellular context. Through the use of a custom peptide array, we determined that Cys^13^ is essential for RGS2 binding to FBXO44. Peptide microarray is a powerful method to probe (PPI) interfaces *in vitro* and is commonly used to determine antibody epitopes to identify proteins responsible for immune response to viral infections, measure the specificity of post-translational modification by specific proteins, and identify peptide inhibitors of PPIs, among other applications (37, 38, 39, 40, 41). The preliminary *K*_*D*_ determined through SPR confirmed that the FBXO44^FBA^–RGS2^5–16^ peptide interaction was satisfactorily of high affinity to make the peptide microarray a viable approach.

The results of this peptide array indicated that Met^5^–Val^9^ are not essential for the RGS2–FBXO44 interaction *in vitr*o. While we expected amino acid substitutions in His^11^–Met^16^ to impact RGS2 binding to FBXO44 more than those in Met^5^–Val^9^, we did not expect the N-terminal half of the peptide to be as amenable to substitution as our results indicate. Previous research indicates that while scrambling the sequence of Phe^6^–Val^9^ has no effect on RGS2 protein half-life, substituting a negatively charged Asp residue extends RGS2 half-life (31). While this study did not look at the RGS2–FBXO44 interaction, an extended protein half-life is indicative of impaired degradation, likely at the E3–substrate interface. It is possible that FBXO44 interacts slightly differently with the peptide *in vitro* than it does with full-length RGS2 in a cellular environment, explaining why we did not observe impaired binding when substituting Met^5^–Val^9^ with Asp. This could be confirmed in future studies with co-IP of full-length RGS2 and FBXO44.

The most prominent result from the peptide microarray was the finding that Cys^13^ is near essential for FBXO44 interacting with RGS2. When truncating the peptide from the C terminus, all interaction was lost once Cys^13^ was removed (Fig. 2*A*), and substituting Cys^13^ with any other residue virtually abolished the interaction, except when substituted with Lys or Arg (both positively charged amino acids) or Tyr or Trp (both bulky hydrophobic residues) (Figs. 2 and 3*C*). In the case of Tyr and Trp, variation was incredibly high, making it difficult to draw conclusions regarding if these would be well-tolerated substitutions in cells, whereas in the case of the substitutions with Lys and Arg, these were well tolerated in other positions as well. In fact, substituting Arg for any of the His^11^–Met^16^ residues seemed to improve binding, though variation remained high at Cys^13^. This suggested that the FBXO44–substrate interface may possess some negatively charged residues that play a role in stabilizing its interaction with RGS2. This was confirmed by the AlphaFold model (Fig. 4*B*) and supports the idea that FBXO44 binds substrates partly through electrostatic interactions.

To further confirm the role of Cys^13^ in FBXO44-mediated degradation of RGS2, we measured how the C13S mutation alters RGS2 co-IP with FBXO44, as well as RGS2 protein half-life and stability. Indeed, we confirmed that Cys^13^ is essential for the interaction and subsequent proteasomal degradation of RGS2 (Figs. 6*A* and 7). While our peptide array indicated that additional residues (Asp12 and Pro15) participate in binding to FBXO44, mutating these residues did not significantly alter the effects of the proteasome inhibitor MG-132 on RGS2 protein levels (Fig. S4). It is possible that the change in affinity resulting from these mutations is not sufficient to affect FBXO44 association or degradation in cells. Future structural biology studies are needed to confirm the impact of these residues on binding to FBXO44.

While our peptide microarray was focused on identifying residues in RGS2 important for binding FBXO44, our AlphaFold predictions and MD simulations also identified substrate-binding residues in FBXO44. Of particular note, Tyr^234^ and Trp^235^ are predicted to be contact residues. These are located in the β9–β10 loop of the FBA domain and are conserved between FBXO44 and the closely related FBXO2 (35). This was a surprising result, given that FBXO44 does not bind the same substrates as FBXO2 (27), and these residues are responsible for substrate binding in FBXO2. Future structural biology work is needed to confirm the substrate-binding properties of FBXO44. However, we were able to confirm the involvement of Tyr^234^ in binding RGS2 through co-IP (Fig. 6*B*).

AlphaFold-predicted structures have improved much in recent years and have become powerful tools in biology and drug discovery. However, it is important to be cautious when interpreting them. In our case, without a structure of FBXO44 in complex with either the RGS2^5–16^ peptide (or full-length RGS2), we can still utilize the predicted structure as a tool to form hypotheses that can be tested experimentally. Several of the predicted contact residues on FBXO44 match residues previously identified to be unique to FBXO44 or form unique hydrogen bonds between β–β loops, indicating that these residues may play a role in substrate recognition (35). Mutating contact residues to alanine, as initiated in this study, is a first step toward experimentally testing the predicted structure. Another approach we can take is to perform hydrogen–deuterium exchange with FBXO44^FBA^ incubated with the RGS2^5–16^ peptide, which would allow us to confirm the accuracy of the predicted binding site with mass spectrometry.

As described previously, FBXO44 does not bind the same substrates as its closely related family members, such as FBXO2 (27). Apart from RGS2, the only other FBXO44 substrate identified thus far is BRCA1 (25), as well as the recently discovered substrate pregnane X receptor (26). The interaction interface between FBXO44 and its other substrates has not yet been elucidated, and the sequence in RGS2 that we have determined serves as the degron for FBXO44 is not conserved in these other two substrates. Thus, it remains an open question how (i) FBXO44 recognizes its substrates and (ii) what the consensus degron sequence is that renders a substrate to be recognized by FBXO44.

Altogether, we have determined that Cys^13^ of RGS2 is essential for FBXO44 binding. Substitution of Cys^13^ not only inhibits FBXO44^FBA^ binding *in vitro* but also stabilizes RGS2 in cells and protects it from proteasomal degradation. Further, our results indicate that the RGS2–FBXO44 interface possesses a negative charge, as both Arg and Lys substitutions enhance association with FBXO44^FBA^ *in vitro*, and AlphaFold 3 predicts the RGS2^5–16^ peptide binding near negatively charged residues on FBXO44 *in silico*. In addition, we show that while FBXO44 does not bind the same substrates as its closely related family members, it binds its substrates through residues conserved between these F-box proteins. There may be additional mechanisms important for FBXO44 (or FBXO2) substrate recognition not yet known, which account for the differences in substrate selectivity.

The studies undertaken here represent a first step toward structural determinants of FBXO44 substrate recognition. Altogether, this will aid in the design of inhibitors of the RGS2–FBXO44 interaction. These would serve as molecular probes and possibly future therapeutics to stabilize RGS2 protein in pathologies associated with low RGS2 protein levels.

## Experimental procedures

### Materials

All chemicals were purchased from MilliporeSigma, unless otherwise stated. The peptide microarray (microarray identifier: 003411_06), incubation tray, and Rockland blocking buffer (Rockland Immunochemicals; MB-070) were purchased from PEPperPRINT GmbH. RGS2^5–16^ and RGS4^1–11^ peptides were purchased from GenScript.

### DNA constructs

FLAG-FBXO44^FBA^ was cloned into a modified pET28 vector with an N-terminal 6xHis-tag, a maltose-binding protein (MBP) fusion tag, and a tobacco etch virus (TEV) protease site. FLAG-FBXO44, RGS2-HA, and RGS2-FLAG were previously used (13, 28). FLAG-FBXO44^Y234A^, RGS2^C13S^-FLAG, and RGS2^C13S^-HA were created using QuikChange II site-directed mutagenesis according to the manufacturer's instructions.

### Antibodies

Monoclonal mouse anti-FLAG DyLight800 antibody and anti-HA (12CA5)-DyLight680 control antibody were purchased from PEPperPRINT GmbH. Rabbit anti-FLAG antibody, monoclonal mouse anti-FLAG M2 antibody, mouse anti-β-actin (ACTB) antibody, rabbit anti-HA antibody, and rabbit IgG were purchased from MilliporeSigma. Rat anti-HA high affinity was purchased from Roche. IRDye 800 CW goat anti-mouse IgG secondary antibody and IRDye 680RD goat anti-rabbit IgG secondary antibodies were purchased from Li-Cor Biosciences.

### FBXO44^FBA^ expression and purification

pET28-His-MBP-TEV-FLAG-FBXO44^FBA^ plasmid was transformed into competent *Escherichia coli* (Rosetta BL21 (λDE3); Novagen) cells. Positive transformants were selected with 50 μg/ml kanamycin and 34 μg/ml chloramphenicol on LB media agar plates. Individual colonies were inoculated in 10 ml LB for overnight growth. LB cultures (1 l) were inoculated with the 10 ml cultures and grown at an absorbance of 0.6 at 600 nm at 37 °C. The 1 l cultures were cooled to 20 °C before recombinant protein induction with 0.5 mM IPTG for 20 h. Cell pellets were frozen at −80 °C. Cell pellets were resuspended in lysis buffer (20 mM Tris–HCl, pH 7.2, 200 mM NaCl, 20 mM imidazole, 0.5 mM EDTA, 3 mM 2-mercaptoethanol, 1× cOmplete, EDTA-free Protease Inhibitor Cocktail [Roche], 10 μg/ml deoxyribonuclease I [Alfa Aesar]) and then lysed using a microfluidizer. The soluble protein supernatant was isolated by ultracentrifugation at 91,000*g* for 1 h at 4 °C. Soluble protein was injected onto a 5 ml HiTrap Chelating HP column (GE Healthcare) and pre-equilibrated in binding buffer (20 mM Tris–HCl, pH 7.2, 200 mM NaCl, and 20 mM imidazole) at 4 °C. The column was washed with binding buffer, and the bound protein was eluted with a step gradient of elution buffer (20 mM Tris–HCl, pH 7.2, 200 mM NaCl, and 500 mM imidazole). Fractions containing the induced protein were combined and dialyzed overnight at 4 °C in storage buffer (20 mM Tris–HCl, pH 7.2, 200 mM NaCl, and 20% v/v glycerol). Aliquots were flash frozen in liquid nitrogen and stored at −80 °C. Pure His-MBP-TEV-FLAG-FBXO44^FBA^ protein was thawed and digested with TEV protease (1:25 mg TEV protease to milligrams of purified protein) in storage buffer containing 40 mM imidazole and 2 mM 2-mercaptoethanol at 4 °C overnight. Cleaved protein was injected onto a pre-equilibrated HiTrap Chelating HP column (GE Healthcare) in binding buffer. Column flow-through was collected to isolate the cleaved, FLAG-FBXO44^FBA^, whereas the TEV protease, uncleaved protein, and His-MBP-tagged fragments were bound. Monomeric FLAG-FBXO44^FBA^ was isolated by injection onto a HiLoad 16/600 Superdex 200 pg (GE Healthcare) gel filtration column equilibrated at 4 °C in size-exclusion chromatography/SPR buffer (20 mM Tris–HCl, pH 7.2, 200 mM NaCl).

### Dot blot

Peptides (RGS2^5–16^ or RGS4^1–11^) were diluted in PBS and then spotted onto Immobilon-FL PVDF membrane (Millipore) and allowed to air-dry overnight at room temperature. Membrane was blocked for 1 h and then incubated in 2 μg/ml FBXO44^FBA^ in blocking buffer overnight at 4 °C. The membrane was then incubated for 2 h at 4 °C in Rabbit anti-FLAG antibody (1:1000 dilution) and finally incubated for 1 h at 4 °C with goat anti-rabbit IRDye 680RD secondary antibody (1:25,000 dilution). Following each antibody incubation, membranes were washed four times in PBS with 0.1% Tween-20. Membranes were imaged using a Li-Cor Odyssey CLx imager.

### Surface plasmon resonance

RGS2^5–16^ peptide was immobilized onto a CM5 chip (Cytiva; BR100012) using amine coupling (Cytiva; BR100557) following the manufacturer's protocol. RGS2^5–16^ peptide (150 μg/ml) in 10 mM NaOAc, pH 4.0, was loaded into the Biacore X100 sample rack. FC1 was immobilized with blank buffer, and FC2 was immobilized with RGS2^5–16^ peptide using 0.4 M 1-ethyl-3-(3-dimethylaminopropyl)carbodiimide, 0.1 M *N*-hydroxysuccinimide, and 1 M ethanolamine. FC1 reached 272 response units (RUs) compared with baseline, whereas FC2 reached 2498 RU, indicating successful immobilization. Using the chip with Fc1-blank and Fc2 immobilized with 2498 RU of RGS2 peptide, FBXO44^FBA^ was flowed over the chip using the recommended flow rates and a contact time of 60 s, starting at the lowest concentration of 0.29 μM, followed by regeneration using 50 mM NaOH. This was repeated through to the highest concentration at 75 μM. The RU at equilibrium was subtracted from the nonspecific binding detected in Fc1 and measured for each concentration.

### Peptide microarray

The microarray slide was seated into the incubation tray according to PEPperPRINT's instructions. The array well was then incubated in washing buffer (PBS) with 0.005% Tween (Sigma; P1379, pH 7.4) at room temperature for 15 min on an orbital shaker. Washing buffer was aspirated, and the array was then incubated with Rockland blocking buffer at room temperature for 30 min on an orbital shaker. Blocking buffer was then aspirated, and the array was incubated in staining buffer (washing buffer with 10% Rockland blocking buffer) at room temperature for 15 min on an orbital shaker. Staining buffer was aspirated, and purified FBXO44 FBA domain diluted to 2 μg/ml in staining buffer was added. The array was then placed on an orbital shaker overnight at 4 °C. The FBA domain solution was removed, and an array was washed three times with washing buffer. After the third array was incubated in staining buffer for 15 min at 4 °C on an orbital shaker. The array was then incubated with antibody dilution (anti-FLAG DyLight800 and anti-HA DyLight680, both diluted 1:2000 in staining buffer) for 45 min at 4° C on an orbital shaker. The array was then washed three times with washing buffer. After the last wash, the incubation tray was disassembled and the slide gently submerged in dipping buffer (1 mM Tris–HCl, pH 7.4) three times and then dried with pressurized argon. Arrays were imaged using a Li-Cor Odyssey CLx imager. Quantification was performed with Innopsys Life Sciences MAPIX software using the Grid Alignment file provided by PEPperPRINT.

### Cell culture and transfections

Cells were maintained in a humidified incubator at 37 °C with 5% CO_2_. HEK-293T cells were obtained from the American Type Culture Collection (ATCC; CRL-1573). The American Type Culture Collection cell lines are subjected to comprehensive and repeated authentication and contamination checks, ensuring confirmation of cell line identity and ensuring that it is free of contamination prior to supplying it to users. Cells underwent periodical (every 6 months) mycoplasma testing to ensure cell lines are not compromised. Cells were cultured in Dulbecco's modified Eagle's medium (Gibco, catalog no.: 11995) and supplemented with 10% fetal bovine serum (Gibco, catalog no.: 16000). Cells were transfected with DNA plasmids using Lipofectamine 3000 (Invitrogen) under reduced serum conditions in Opti-MEM (Gibco, catalog no.: 31985). Media were changed to Dulbecco's modified Eagle's medium supplemented with 0.5% fetal bovine serum after 4 to 6 h, and cells were harvested for experiments 24 h after transfection. siRNA transfections were performed under reduced serum conditions in Opti-MEM 24 h prior to DNA transfections at 40% to 60% confluency. Cells were transfected with siGENOME SMART-POOL siRNA (FBXO44, M-019201-01-0005; Nontargeting, D-001206-13-05) from Dharmacon (Revvity) using Lipofectamine RNAiMAX (Invitrogen). Experiments were performed 72 h after transfection.

### Coimmunoprecipitation

Cells were washed with PBS and then lysed in radioimmunoprecipitation assay buffer containing protease inhibitors (50 mM Tris–HCl, pH 7.4, 150 mM NaCl, 0.25% w/v deoxycholate, 1 mM EDTA, 1% NP-40, cOmplete Protease Inhibitor Cocktail EDTA-free) for 30 min on ice with occasional vortexing. Lysate was centrifuged, and the supernatant was transferred to clean tubes. Samples were incubated with 40 μl protein A agarose beads (Roche) for 30 min on a rotator at 4 °C. Samples were then centrifuged, and the supernatant was used to determine total protein concentration with the Pierce BCA Protein Assay Kit (Thermo Scientific). Total protein (500 μg) in a volume of 500 μl was used for each IP reaction (1 mg/ml). Thirty microliters were removed from each sample for total input. Three microliters of rabbit IgG antibody (Sigma; catalog no.: 12370) were added to the negative sample, 3 μl of rabbit anti-HA antibody (Sigma; H6908) and 40 μl of protein A agarose beads were added to each sample prior to incubation on a rotator at 4 °C for 2 h. Samples were centrifuged, and the supernatant was removed. Samples were washed three times with 1 ml PBS. Bound proteins were eluted from beads at 95 °C in 55 μl protein sample loading buffer (Li-Cor Biosciences).

### CHX chase assay

HEK-293T cells in 12-well plates, transiently transfected with either RGS2^WT^ or RGS2^C13S^, were treated with CHX (50 μM) at indicated time points. Cells were harvested as described later and subjected to SDS-PAGE and Western blot.

### Preparation of cell lysates

Cells were harvested on ice in lysis buffer containing protease inhibitors (50 mM Tris–HCl [pH 7.4], 5 mM NaCl, 0.5 mM EDTA, 1% Triton X-100, 10% cOmplete Protease Inhibitor Cocktail EDTA-free). Lysates were bath sonicated for 10 min at 4 °C, centrifuged at 6000*g* for 3 min, and the supernatant was used for SDS-PAGE and immunoblotting. Total protein concentration was determined using the Pierce BCA Protein Assay Kit. Samples were diluted to 1 μg/μl in protein sample loading buffer (Li-Cor Biosciences).

### SDS-PAGE and Western blot

Equal amounts of protein in each lane were resolved on a 12% SDS-PAGE gel for 1 h at 160 V. Samples were transferred to an Immobilon-FL PVDF membrane (Millipore) and subjected to Western immunoblot analysis using Li-Cor blocking buffer for both blocking and antibody diluents. Membranes were blocked for 1 h, then incubated for 2 h in primary antibodies as described under “Antibodies,” and finally incubated for 1 h with IRDye secondary antibodies directed at the species of the primary antibody. Following each antibody incubation, membranes were washed four times in PBS with 0.1% Tween-20. Membranes were imaged using an Azure600 imaging system (Azure Biosystems).

### AlphaFold multimer

Amino acid sequences for human FBXO44 (UniProt: Q9H4M3-1) and the RGS2^5–16^ peptide (MFLAVQHDCRPM) were inputted into the AlphaFold 3 server (https://alphafoldserver.com/) (42). Five models were generated (Fig. S1), and the best model, determined collectively by predicted template modeling score, pLDDT score, predicted aligned error score, and agreement with our microarray results, was loaded into ChimeraX to obtain images (Fig. S2). Contact residues were determined using the Contacts tool of ChimeraX using van der Waals overlap of ≥-0.40 Å. The modeled structure was deposited in ModelArchive (43) at https://www.modelarchive.org/doi/10.5452/ma-qnfim.

### MD simulations

The top five conformations generated with AlphaFold Multimer were selected for simulation, and four independent simulations were performed for each starting conformation. To prepare the systems for simulation, first the flanking residues were modeled and added to RGS2^5–16^ to generate RGS2^1–22^ using Chimera (44). Following the addition of these residues, 1000 steps of steepest descent energy minimizations of the PPIs were carried out in Chimera to reduce side-chain clashes. These minimized structures were then prepared and run with all-atom MD using GROMACS 2021.2 (45). The simulation workflow included system preparation, energy minimization, solvation and ion addition, thermal and pressure equilibration in constant volume and temperature (NVT) and constant pressure and temperature (NPT) ensembles, and production MD runs in the NPT ensemble. The AMBER99SB-ILDN force field (46) was used to parameterize proteins. Water molecules were modeled using the TIP3P water model.

For each simulation, the simulation box is a triclinic box with a minimum distance of 1.0 nm between the solute and the box edge and periodic boundary conditions. Solvation was performed by filling the box with TIP3P water. The system was then neutralized and adjusted to a physiological ionic strength (0.15 M) by adding Na^+^ and Cl^-^ ions using the gmx genion command (45), with replacement of solvent molecules.

To prepare the systems for production runs, first energy minimization was performed until the maximum force on any atom was less than 1000.0 kJ mol^−1^ nm^−1^ or until 50,000 steps were completed. Verlet cutoff and particle-mesh Ewald electrostatics were employed with 1.0 nm cutoffs. Subsequently, equilibration under NVT was performed for 500 ps using the leap-frog integrator with a time step of 2 fs. The V-rescale thermostat maintained a temperature of 300 K with a coupling time constant τ = 0.1 ps. Position restraints were applied to the heavy atoms of the protein. Next, each system was further equilibrated for 300 ps under NPT conditions using the Parrinello–Rahman barostat with a reference pressure of 1.0 bar with isotropic coupling and τ = 2.0 ps. Temperature settings and thermostat parameters matched the NVT phase.

The production simulations were conducted under NPT conditions for 10 ns with a 2 fs timestep. Electrostatics were handled with particle-mesh Ewald, and van der Waals and Coulomb interactions were truncated at 1.0 nm using the Verlet scheme. Coordinates and energies were saved every 10 ps for analysis. LINCS constraints were applied to all covalent bonds involving hydrogen. For analysis of the simulations, rotational and translational motions were removed, and periodic boundary conditions were unwrapped. All scripts to prepare the simulations, Gromacs commands used, and simulation files are available open-source (47).

### Analysis of MD simulations

First, the RMSD of the peptides was calculated and plotted in comparison to the starting simulation structure using Gromacs utilities (45). The RMSD over time showed a burn-in period of less than 500 frames. Protein RMSDs were plotted as averages and variances over trials for each starting conformation. Simulation burn-in frames were removed from subsequent analysis. Code to generate RMSD plots is available open-source (47). Next, the average frequency over MD frames of forming a contact between residues in the PPIs was calculated over trials for all conformations. Two residues were treated as being in contact if the α-carbons were within 0.8 nm of each other. The contact scores were normalized from 0 to 1, where a score of 0 indicates two residues were never in contact, and a score of 1 indicates that two residues were in contact continuously over all simulation trials. The code to specify the parameters used and generate plots, *plot_contacts.py*, and the code to calculate average contacts over variable simulation trials, *contact_analysis.py*, are available open-source (47).

## Statistical analysis

Western blot images were quantified using Image Studio software (Li-Cor Biosciences). The intensity of bands for the protein of interest was normalized to β-actin as a loading control. The intensity of bands in co-IP experiments was normalized to the input level of the same protein. All data were analyzed using GraphPad Prism 10.0 (GraphPad). Datasets with two groups were analyzed using Student's *t* test. Datasets with three or more groups were analyzed with one-way or two-way ANOVA, depending on the nature of the groups as indicated in each figure. All experiments were run at least three times, unless otherwise indicated in the figure caption. Data are presented as mean ± SD with a *p* < 0.05 considered significant.

## Data availability

All data are contained within the article, except for the following: The AlphaFold structure was deposited in ModelArchive https://doi.org/10.5452/ma-qnfim; protein simulations and analysis code and data are available open-source through Zenodo: https://doi.org/10.5281/zenodo.15733902. Any inquiries regarding the data should be directed to the corresponding author, Benita Sjögren, jsjogren@uci.edu.

## Supporting information

This article contains supporting information.

## Conflict of interest

The authors declare that they have no conflicts of interest with the contents of this article.
