# Supplementary material for: Systematic analysis of the RGS2 degron reveals characteristics of substrate recognition by the F-box protein FBXO44
Source: J Biol Chem. 2025 Sep 22;301(11):110757. doi: 10.1016/j.jbc.2025.110757 (PMC12554172; doi:10.1016/j.jbc.2025.110757)
Supplement: Supporting information [file mmc1.pdf]

Supporting information

Systematic analysis of the RGS2 degron reveals characteristics of substrate recognition by the F-box protein FBXO44

Harrison J. McNabb[1], Eugene Cho[2], Mary Pitman[2], Phillip S. Rushton[1], David Mobley[2] and Benita Sjögren[1,2, 3]*

Contents

Table S1. Statistical Summary of Microarray Scans of the $RGS2^{5\text{-}16}$-$FBXO44^{FBA}$ interaction S2

Figure S1. SPR sensogram of $FBXO44^{FBA}$ binding immobilized $RGS2^{5\text{-}16}$ peptide..................... S4

Figure S2. Overlay of the 5 best fit AlphaFold models for FBXO44-RGS2 peptide interaction. .. S5

Figure S3. Quality control for AlphaFold 3 model S5

Figure S4. Effect of RGS2 mutations on protein stability. S6

Table S1. Statistical Summary of Microarray Scans of the RGS2$^{5\text{-}16}$-FBXO44^{FBA} interaction. Amino acid substitutions not presented in the table displayed no statistical significance.

Original residue	Substitution	Mean % difference from parent peptide	Adjusted *P*-value	*P*-value summary
A8	R	27.84	0.0096	**
H11	R	41.19	<0.0001	****
D12	C	43.97	<0.0001	****
D12	F	34.22	0.0005	***
D12	G	28.74	0.0065	**
D12	H	37.64	<0.0001	****
D12	I	42.93	<0.0001	****
D12	K	124.2	<0.0001	****
D12	L	31.38	0.0019	**
D12	M	42.32	<0.0001	****
D12	N	55.82	<0.0001	****
D12	P	66.54	<0.0001	****
D12	Q	59.62	<0.0001	****
D12	R	184.9	<0.0001	****
D12	S	59.59	<0.0001	****
D12	T	32.23	0.0013	**
D12	V	40.66	<0.0001	****
D12	W	48.95	<0.0001	****
D12	Y	31.8	0.0016	**
C13	A	-72.59	<0.0001	****
C13	D	-75.39	<0.0001	****
C13	E	-87.12	<0.0001	****
C13	F	-82.91	<0.0001	****
C13	G	-89.67	<0.0001	****
C13	H	-83.62	<0.0001	****
C13	I	-87.24	<0.0001	****
C13	K	-53.92	<0.0001	****
C13	L	-88.83	<0.0001	****
C13	M	-88.01	<0.0001	****
C13	N	-85.38	<0.0001	****
C13	P	-84.68	<0.0001	****
C13	Q	-77.02	<0.0001	****
C13	R	35.61	0.0002	***

C13	S	-87.05	<0.0001	****
C13	T	-86.63	<0.0001	****
C13	V	-88.12	<0.0001	****
C13	W	-46.69	<0.0001	****
C13	Y	-42.98	<0.0001	****
R14	A	-27.71	0.0101	*
R14	W	32.54	0.0011	**
P15	A	-27.06	0.0132	*
P15	C	26.71	0.0152	*
P15	D	31.74	0.0016	**
P15	G	-30.63	0.0028	**
P15	K	52.32	<0.0001	****
P15	R	158	<0.0001	****
M16	A	-33.89	0.0006	***
M16	C	30.03	0.0037	**
M16	D	31.28	0.002	**
M16	G	-27.86	0.0095	**
M16	K	38.94	<0.0001	****
M16	R	101.4	<0.0001	****

*$P<0.05$; **$P<0.01$; ***$P<0.001$; ****$P<0.0001$ using two-way ANOVA followed by Dunnet's *post hoc* test for pairwise comparison within rows.

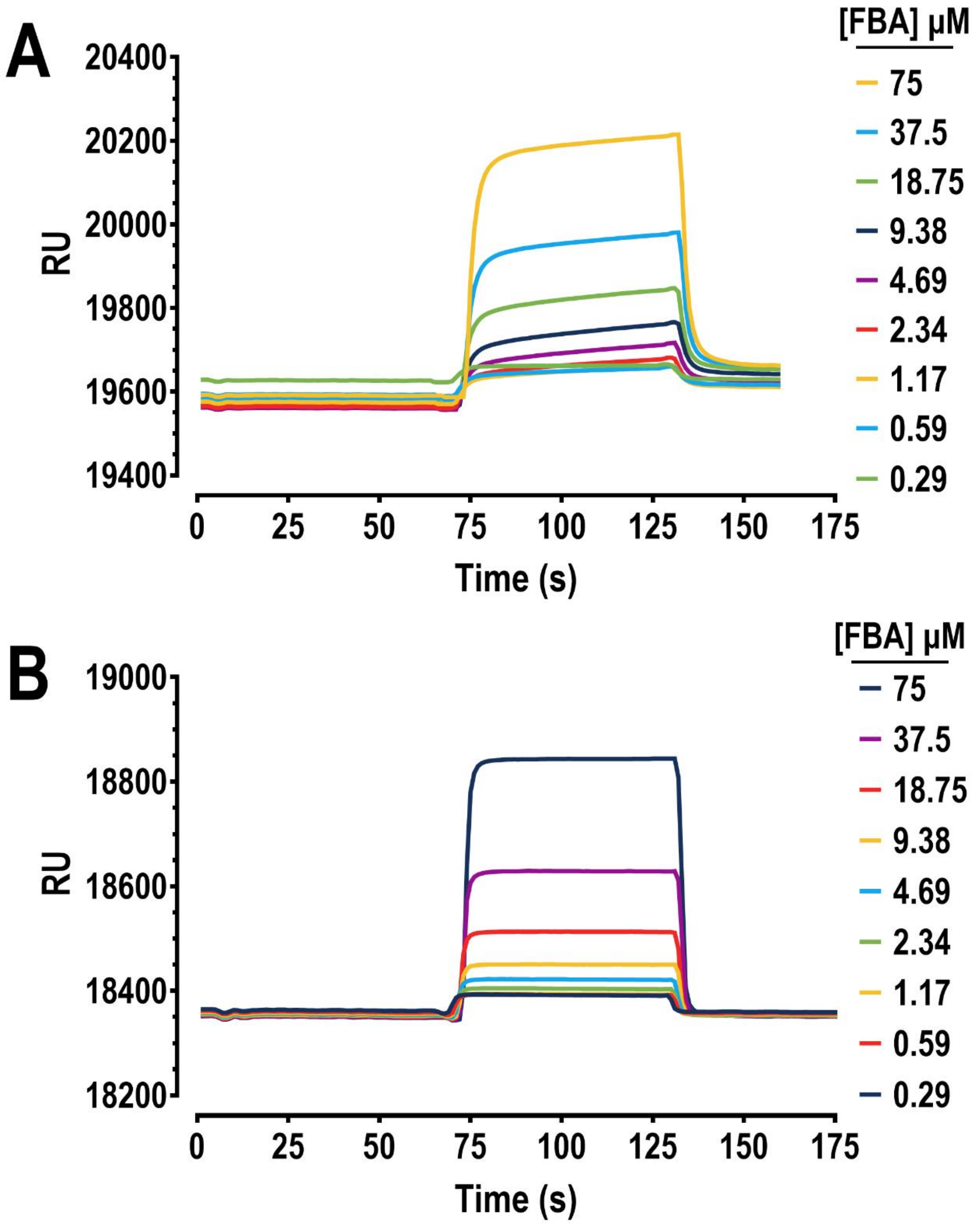

Figure S1. SPR sensogram of FBXO44^{FBA} binding immobilized RGS2$^{5\text{-}16}$ peptide. Fc1 was immobilized with blank buffer and Fc2 was immobilized with RGS2$^{5\text{-}16}$ peptide using 0.4 M EDC, 0.1 M NHS and 1 M ethanolamine. FBXO44^{FBA} was flowed over the chip using the recommended flow rates and a contact time of 60 s, starting at the lowest concentration of 0.29 μM followed by regeneration using 50 mM NaOH. This was repeated through to the highest concentration at 75 μM. **A.** Sensogram of total FBXO^{FBA} binding to RGS2$^{5\text{-}16}$ in Fc2. **B.** Sensogram of non-specific FBXO^{FBA} binding to the blank Fc1. The RU at equilibrium was subtracted from the non-specific binding detected in Fc1 and measured for each concentration, as presented in **Fig. 1** of the main manuscript.

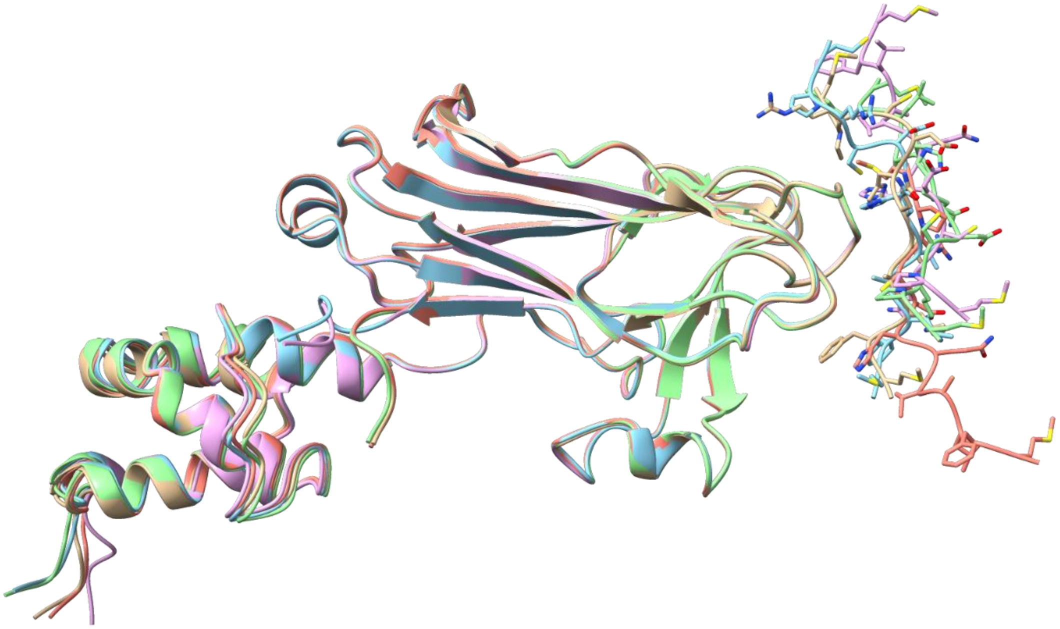

Figure S2. Overlay of the 5 best fit AlphaFold models for FBXO44-RGS2 peptide interaction. Aligned overlay of the 5 AlphaFold 3 models. The model colored in light blue was deemed best as determined by pLDDT, Predicted Aligned Error (PAE) map (**Fig. S2**), and our microarray results.

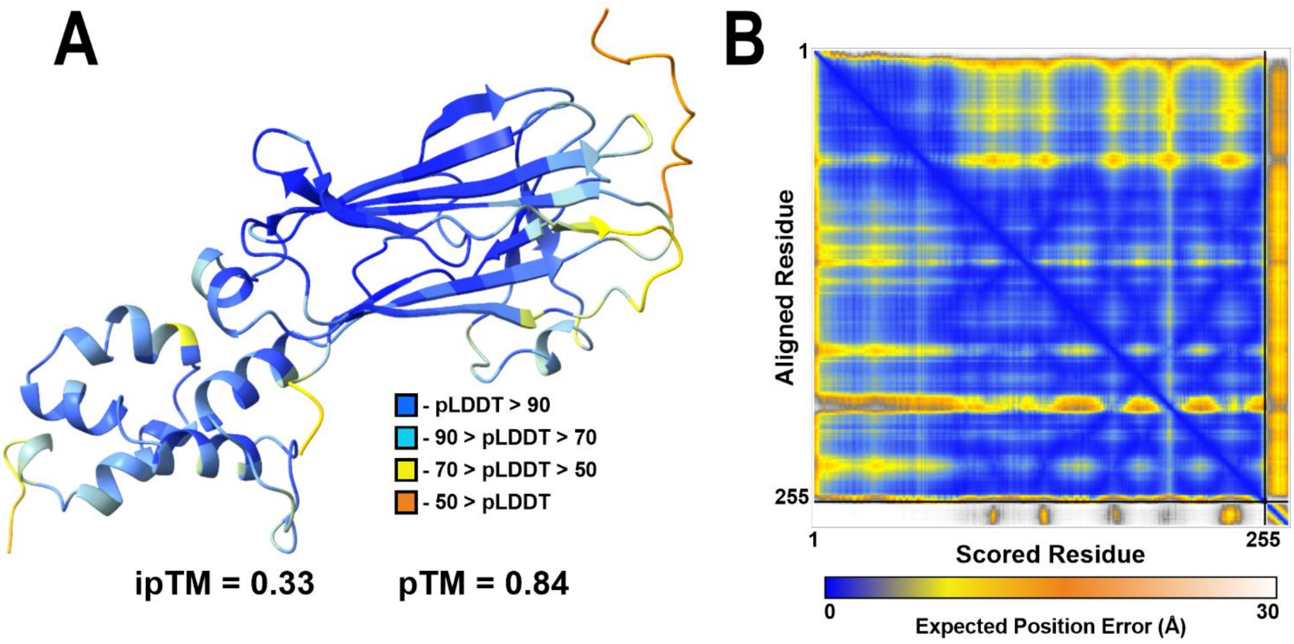

Figure S3. Quality control for AlphaFold 3 model. A. pLDDT Map of predicted structure with provided scale, interface predicted template modeling (ipTM) and predicted template modeling (pTM) scores. **B.** PAE map of the aligned structures with the N- and C-terminal residues of FBXO44 indicated on the axes.

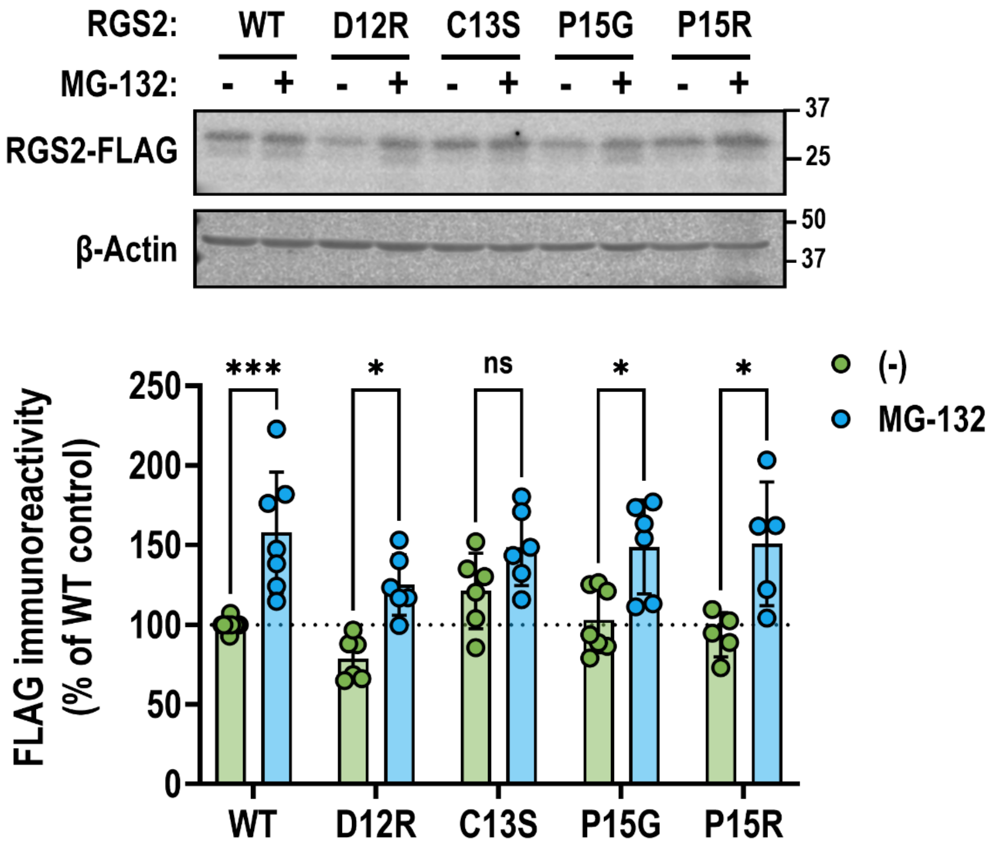

Figure S4. Effect of RGS2 mutations on protein stability. Representative western blot and quantification of five or six independent experiments (varies by mutation). The proteasome inhibitor MG-132 (10 μM, 4 h) significantly increases RGS2^{WT} protein levels, as well as all tested mutants, except RGS2^{C13S}. D12R and P15R were predicted to destabilize RGS2, due to increased binding to FBXO44 in the peptide microarray. Although there was a trend towards decreased expression for D12R, this decrease was not statistically significant. *P<0.05; ***P<0.001 using two-way ANOVA followed by Tukey's *post hoc* test for pairwise comparisons
